# JARID1B promotes colorectal cancer proliferation and Wnt/β-catenin signaling via decreasing CDX2 level

**DOI:** 10.1186/s12964-020-00660-4

**Published:** 2020-10-27

**Authors:** Da Huang, Fan Xiao, Haibin Hao, Fuzhou Hua, Zhenzhong Luo, Zhaoxia Huang, Qing Li, Sha Chen, Xiuzhi Cheng, Xinyue Zhang, Weilan Fang, Xiaoyun Hu, Fanrong Liu

**Affiliations:** 1 Department of Thyroid Surgery, Second Affiliated Hospital of Nanchang University, Nanchang, China; 2 Jiangxi Province Key Laboratory of Molecular Medicine, Nanchang, China; 3 Department of Anesthesiology, Second Affiliated Hospital of Nanchang University, Nanchang, China; 4 Department of General Surgery, Second Affiliated Hospital of Nanchang University, Nanchang, China; 5 Center for Education Evaluation, Nanchang Normal University, Nanchang, China; 6 Department of Pathology, Second Affiliated Hospital of Nanchang University, Nanchang, China

**Keywords:** Colorectal cancer, Cell proliferation, JARID1B, CDX2, H3K4me3, Wnt/β-catenin

## Abstract

**Background:**

Jumonji AT-rich interactive domain 1B(JARID1B) has been shown to be upregulated in many human cancers and plays a critical role in the development of cancers cells. Nevertheless, its functional role in colorectal cancer (CRC) progression is not fully understood.

**Methods:**

Herein, JARID1B expression levels were detected in clinical CRC samples by western blotting and qRT-PCR. DLD-1 cells with JARID1B knockdown or overexpression by stably transfected plasmids were used in vitro and in vivo study. Colony formation, 5-ethynyl-20-deoxyuridine (EdU) and Real Time Cellular Analysis (RTCA) assays were used to detect cell proliferation and growth. Transcriptome and CHIP assays were used to examine the molecular biology changes and molecular interaction in these cells. Nude mice was utilized to study the correlation of JARID1B and tumor growth in vivo.

**Results:**

Here, we first observed that JARID1B was significantly upregulated in CRC tissue compared to adjacent normal tissues. In CRC patients, JARID1B high expression was positively relation with poor overall survival. Multivariate analyses revealed that high JARID1B expression was an independent predictive marker for the poor prognosis of CRC. In addition, we found that JARID1B promoted CRC cells proliferation by Wnt/β-catenin signaling pathway. Further studies demonstrated CDX2 as a downstream target of JARID1B, and our data demonstrated that CDX2 is crucial for JARID1B -mediated Wnt/β-catenin signaling pathway. Mechanistically, we demonstrated that JARID1B regulated CDX2 expression through demethylation of H3K4me3.

**Conclusions:**

CDX2 inhibited by JARID1B-derived H3K4me3 methylation promoted cells proliferation of CRC via Wnt/β-catenin signaling pathway. Therefore, our studies provided a novel insight into the role of JARID1B in CRC cells proliferation and potential new molecular target for treating CRC.

Video abstract

**Graphical abstract:**

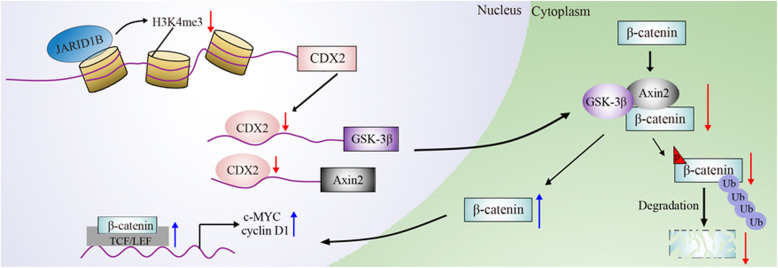

**Supplementary information:**

The online version contains supplementary material available at 10.1186/s12964-020-00660-4.

## Background

Colorectal cancer (CRC) is the most common cancer with high morbidity worldwide and is the third leading cause of cancer-related death in men and the second leading cause in women [1, 2]. In recent decades, although diagnosis and treatment techniques have improved and new cancer therapeutics, including molecular targeted therapies and immunotherapies, have occurred, cancer prognosis is still unsatisfactory because of the dysregulation of cell death and proliferation mechanisms in cancer [3, 4]. Because the cell proliferation and metastasis of CRC are the most common causes of death, exploring the molecular mechanism of CRC cell proliferation is a vital issue in the treatment of CRC [5]. Moreover, epigenetic regulation of gene expression has been shown to provide new insights into the pathogenesis of CRC [6]. In particular, methylation and demethylation of histone lysine residues act as transcriptional switches for gene expression under physiological and disease conditions [7]. In this study, we showed that a new target related to histone methylation modification can regulate CRC cell proliferation.

JARID1 proteins are histone demethylases that regulate the fate of normal cells and contribute to malignant transformation [8]. The JARID1 family members include JARID1A, JARID1B, JARID1C and JARID1D [9, 10]. JARID1B (also known as KDM5B) has been studied mostly so far. JARID1B was originally considered a transcription inhibitor to regulate the cell cycle, cell differentiation and cell proliferation [11]. JARID1B can specifically remove H3K4 trimethylation and inhibit relative gene transcription [11]. Recently, there has been an increasing number of studies on JARID1B in malignant tumours. JARID1B is an oncogenic epigenetic factor overexpressed in different types of cancers, such as breast cancer, lung cancer, prostate cancer, osteosarcoma, oral cancers, melanoma, glioma, hepatocellular carcinoma, gastric cancer and pancreatic cancer [12, 13]. The depletion of JARID1B has been shown to specifically inhibit H3K4 demethylation and suppress CRC cell growth [14]. Nevertheless, the clinical significance of JARID1B expression levels in CRC clinical samples has not been studied in detail, and its specific mechanism of action in the process of CRC progression is still unclear.

The Wnt family consists of 19 secretory cysteine-rich glycoproteins that all play principal regulatory roles in many developmental and biological processes, such as cell fate specification, proliferation, migration and asymmetric cell division [15]. Furthermore, Wnt/β-catenin signalling plays important roles in tissue maintenance and regeneration [16]. Recently, emerging evidence links the biological function of Wnt/β-catenin signalling to tumorigenesis and development [17]. At present, the molecular mechanism of tumorigenesis and development related to the activation of Wnt signalling, which increases β-catenin levels, represents a common pathway in Wnt signalling activation, that is, to enhance the translocation of β-catenin into cell nuclei where it binds to transcription factors of the TCF/LEF family. The β-catenin-TCF/LEF complex then induces transcription of downstream target genes in cancer, such as c-MYC and cyclin D1 [18]. Recent studies have demonstrated that abnormal activation of Wnt/β-catenin signalling has often been reported in colorectal cancer (CRC). For example, activation of the Wnt signalling pathway is required for tumour growth in advanced CRC, and activation of Wnt signalling to β-catenin contributes to the development of CRC [19, 20]. However, the regulatory mechanisms of abnormal activation of Wnt/β-catenin signalling in CRC are not yet clear.

In the present study, we found that JARID1B was elevated in CRC tissues compared with normal colonic tissues using quantitative reverse transcription (qRT)-PCR, western blot and immunohistochemistry. JARID1B expression was correlated with patient survival time. Furthermore, JARID1B promoted CRC cell proliferation in vivo and in vitro. In addition, our results further show that JARID1B regulates Wnt/β-catenin signalling to influence CRC cell proliferation. Additionally, we demonstrated that JARID1B significantly inhibited CDX2 expression in CRC, and CDX2 is crucial for JARID1B-mediated Wnt/β-catenin signalling in CRC. Finally, our study verified that JARID1B decreases CDX2 expression by demethylating H3K4me3. Therefore, our findings indicate that JARID1B is believed to be a promising target in the treatment of CRC.

## Materials and methods

### Clinical samples

All the specimens of 54 patients from the Second Affiliated Hospital of Nanchang University were diagnosed as CRC by pathological test from June 2018 to December 2018. Total proteins and mRNAs from these patients’ clinical tissues were immediately obtained when tissues was still fresh. Moreover, formalin-fixed paraffin-embedded 130 CRC patients’ tissues from December 2012 to December 2017 were randomly selected to observe the expression levels by immunohistochemistry and their corresponding follow-up data about survival was obtained from the hospital database. Informed consent was obtained from all patients, those patients’ clinical data was recorded during hospitalization and the research program was approved by the Ethics Committee of the Second Affiliated Hospital of Nanchang University.

### EDU assay

According to what the manufacturer’s protocol described, 20 μM BrdU was put into CRC cells for 4 h at 37 °C. Then washed with PBS for three times, the cells were mixed with apollo reaction for 1 h. The cells were stained with 100 ul of Hoechst 33342 (5 μg/ml) for 30 min to visualize the nuclei and observed under a fluorescence microscope (Olympus, Tokyo, Japan) [21].

### Real-time proliferation assay

The xCELLigence real-time cell analysis (RTCA) system (ACEA Bioscience) was used to analyze cell proliferation. Cells were seeded on a 96-well plate (E-plate, Germany). After treated with different approaches for 12 h, the growth rates were recorded every 10 min by the instrument. Under the same xCELLigence RTCA program, continue to monitor the changes of cell index for 36 h. We used mean cell index values in real-time to present cell proliferation changes. For each experiment, we tested three biological replicates.

### In vivo tumorigenicity study

After construction of a DLD-1 cell line that stably interfered with JARID1B expression, 5 × 10^6^ cells in 200 ul of PBS were injected subcutaneously into the flanks of nude mice (male athymic BALB/c nude mice,4–6 weeks). We used the random number table as a random method to determine the experimental animals, making sure more than 40 mices. Five mice were randomly selected every 5 days to get tumor tissues, and then tumor volumes were measured according to the protocol: V = 1/2 (largest diameter) × (smallest diameter)^2^. In vivo imaging showed tumor growth on day 30, and fluorescent pictures were taken. After 40 days, tumors tissues from 5 mices were harvested and individually weighed after the mice were anesthetized. The data was presented as tumor weight (mean ± SD).

### Cell culture

Commercialized CRC cell lines SW620, HCT116, LOVO, SW480 and DLD-1 were obtained from American Type Culture Collection (ATCC, Manassas, VA, USA). SW620 and SW480 were cultured in L-15 medium (Gibco). LOVO was cultured in F12K medium (Gibco). DLD-1 and HCT116 were cultured in DMEM medium (Gibco). All cell lines were cultured at 37 °C, 5% CO_2_ condition. Moreover, 10 uM XVA-939 from American AbMole was used to inhibite β-catenin degradation. Over-expression plasmids and shRNA interference fragments are transfected into cells through lipofectamine 2000 and lipofectamine LTX (Catalog:11668019, Invitrogen, Carlsbad, CA, USA).

### Plasmids and reagents

SiRNA of JARID1B and CDX2 was synthesized by InvivoGen. The target sites of shRNA are detailed in Supplementary Table S1. The stable knockdown and overexpressed JARID1B CRC cells according to the Manufacturer’s protocol. The shJARID1B and shCDX2 of CRC cells was selected based on resistance to hygromycin. The pcDNA3.1(+)-JARID1B-expressing CRC cells were selected using G418. The following reagents were used: Lipofectamine 3000 (Catalog: L3000001, Invitrogen, Carlsbad, CA, USA), dual-luciferaseassay kit (Catalog: E1910, Promega), SimpleChipTM Enzymatic Chromatin IP KIT (Catalog: #9003, CST, USA), EdU kit (Catalog: C10310, RiboBio, China).

### qRT-PCR, western blot analysis and co-immunoprecipitation (co-IP)

qRT-PCR and western blot analysis were made as previously reported [22]. Whether it was tissue or cells, total RNA was extracted by Trizol reagent (Catalog:15596026, Invitrogen, USA) and were quantified by SYBR Green assays with RT primers and SYBR Green from Takara Biotechnology (Catalog: DRR041A, TAKARA, Dalian, China). Human GAPDH was amplified in parallel as an internal control. For western blot, total proteins of clinical samples were obtained through RIPA lysis buffer and prepared cells were harvested by RIPA lysis buffer containing protease inhibitor cocktail (Catalog: P8340-1ML, Sigma-Aldrich). All the proteins were fractionated by 10% SDS-PAGE. The antibodies anti-JARID1B(Catalog: ab198884, 1:2000, abcam), anti-GSK-3β(Catalog: ab93926, 1:1000, abcam), anti-Axin2(Catalog: ab109307, 1:1000, abcam), anti-CDX2(Catalog: ab76541, 1:1500, abcam), anti-c-MYC(Catalog: 10828–1-AP, 1:1000, proteintech), anti-phosphorylation-β-catenin(phospho Y142) (Catalog: ab27798, 1:2000, abcam), anti-β-catenin (Catalog: ab6302, 1:2000, abcam), anti-H3K4me3(Catalog: ab8580, 1:1500, abcam), anti-Histone3(Catalog: ab1791, 1:1500, abcam), anti-ubiquitin (Ub) (Catalog: ab7780, 1:500, abcam) and anti-tubulin (Catalog: 11224–1-AP, 1:1000, proteintech) were used. For co-immunoprecipitation, to deal with cells for 24 h with 10 umol/L MG132, cell lysates were incubated overnight at 4 °C with anti-β-catenin, then conjugated to protein A/G agarose beads while rocking. Immunoprecipitates were washed with washing buffer (50 mM Tris (pH 7.5), 150 mM NaCl, 1% Triton-X), re-suspended in 2 × loading buffer, and resolved by SDS-PAGE followed by immunoblotting analysis.

### Gene set enrichment analysis (GSEA)

GSEA was a statistical analysis method for assessing whether a particular gene set showed statistically significant and consistent differences between two biological states, such as tumor and non-tumor or low-expression and high-expression groups [23]. Subsequently, mRNAs that were differentially expressed in CRC were imported into the GSEA software for Kyoto Encyclopedia of Genes and Genomes (KEGG) (http://www.kegg.jp) assessment. Results from the GSEA software were visualized by the GOplot R software package. The GSEA software used a genome false discovery rate (FDR) of less than 25%, suggesting that FDR < 25% indicated significantly enriched gene sets.

### Luciferase reporter assay

Fragments of the CDX2 were amplified PCR using primers (Supplementary Table S1) and cloned into the luciferase reporter vector pGL3.0-Basic (Catalog: E1751, Promega, Madison, WI, USA) to generate CDX2 promoter reporter constructs. Plasmids containing firefly luciferase reporters and JARID1B plasmids were cotransfected into cells. For the TOP/FOP-Flash reporter assay [24], the TOP/FOP-Flash reporter and JARID1B plasmids were co-transfected into cells. After transfection for 48 h, the cells were harvested for analysis with the Dual-Luciferase Reporter Assay System (Catalog: E1910, Promega, Madison, WI, USA). Luciferase activity was measured using the PerkinElmer EnSpire Multilabel Reader 2300 (PerkinElmer Inc., Waltham, MA, USA). The luciferase intensity was normalized to the Renilla luciferase activity to normalize for transfection efficiency.

### Chromatin immunoprecipitation assay (ChIP)

ChIP assays were performed using the ChIP assay kit (Catalog: 8982, Cell Signaling Technology). Briefly, the cells were fixed with 1% formaldehyde for 10 min at room temperature, then washed with PBS for three times. After a series of processing according to the protocol, Digested DNA fragments was sonicated in the range of 150–300 bp. The immunoprecipitations were mixed with H3K4me3 antibody and agarose. The input and DNA were then subjected to qPCR.

### Statistical analysis

Paired t test for the two groups and one-way ANOVA for more than two groups were used to analyze JARID1B expression data and its relationship with various clinicopathological factors. When data did not follow normal distribution, Mann-Whitney U test between two groups and Kruskal-Wallis H test for three or more groups should be used. Kaplan-Meier analysis and the logrank test were used for survival analysis. Correlation between two continuous values was analyzed by pearson’s correlation. Furthermore, univariate and multivariate analyses were performed using the logistic regression model. All of the data were analyzed using GraphPad Prism and SPSS 22.0. *p*-values of < 0.05 indicated statistically significant changes. Each experimental design included more than three biological repeats.

## Results

### JARID1B was markedly upregulated in CRC tissues and was closely related to CRC progression

**Fig. 1:**
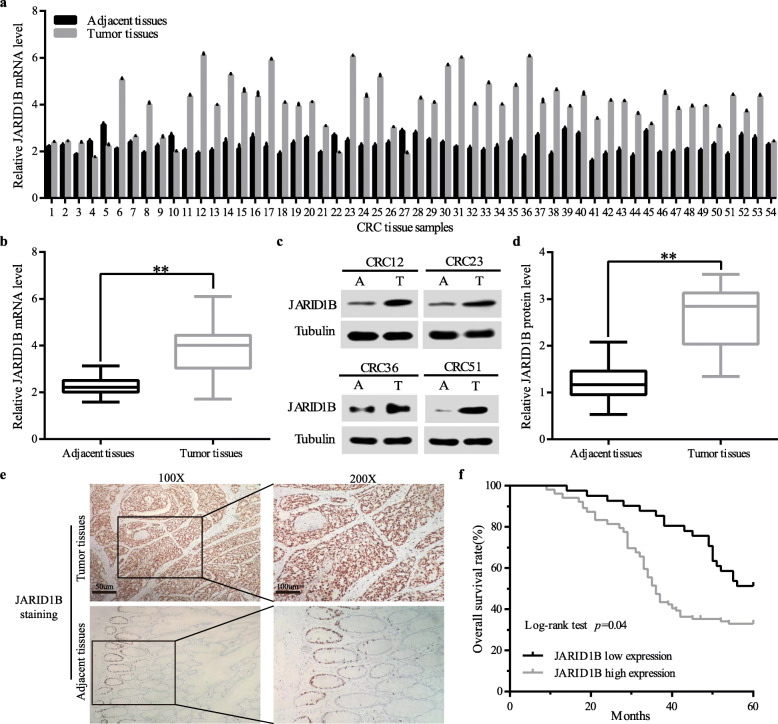
JARID1B overexpression was associated with poor prognosis in patients with CRC. **a** Relative JARID1B mRNA expression levels in fresh 54 paired colorectalcancer and para-carcinoma tissues. **b** JARID1B expression was measured by qRT-PCR in tumor tissues and adjacent tissues. **c** Representative western blotting analysis of JARID1B protein expression (T, tumor tissues; A, adjacent tissues). **d** Quantification of JARID1B protein expression based on western blot analyses in fresh 54 paired CRC and adjacent non-tumor tissues. **e** Representative images and quantification of JARID1B staining in the paired CRC tissues. Scale bar, 50 μm. g Kaplan-Meier survival curves for 130 CRC patients showed JARID1B high expression had poor prognosis. *p < 0.05, **p < 0.01

To investigate the role of JARID1B in the development of colorectal cancer, we investigated JARID1B expression levels in colorectal cancer, and 54 paired fresh CRC tissue samples and matched adjacent non-tumour tissues were used. qRT-PCR revealed that the mRNA expression level of JARID1B was notably elevated in CRC tissue samples compared with the corresponding adjacent tissues (Fig. 1a, b). Furthermore, western blotting showed that JARID1B protein expression in 54 paired CRC tissues was consistent with the qRT-PCR results (Fig. 1c, d). Consistently, immunohistochemistry (IHC) results showed that JARID1B protein was highly expressed in 75.38% (98 of 130) of tissues, while weakly positive staining was observed in the adjacent non-tumour tissues (Fig. 1e). Then, we explored whether JARID1B expression affected clinicopathological parameters. As shown in Table 1, the high JARID1B expression group demonstrated a larger tumour size, higher CEA levels and greater T classification compared with the low JARID1B expression group. Furthermore, the Kaplan-Meier survival curves demonstrated that patients with high JARID1B expression levels in 130 immunohistochemical results had a significantly poorer overall survival than those with low expression levels (Fig. 1f), while univariate and multivariate analysis of overall survival in CRC patients also indicated that high JARID1B expression was a risk factor for 5-year survival (Table 2). Altogether, the findings demonstrated that JARID1B expression is upregulated in CRC and is implicated in the progression of CRC.

**Table 1:** Relationship between JARID1B protein expression and clinicopathological features in CRC patients

Parameters	Total 54^a^	Relative JARID1B protein expression^b^	*P* value
Gender			0.53
Male	36	2.11 ± 0.85	
Female	18	2.34 ± 0.89	
Age (years)			0.71
≤ 60	20	2.14 ± 0.93	
> 60	34	2.22 ± 0.83	
Tumor size (cm)			0.00^**^
< 5	23	1.61 ± 0.80	
≥ 5	31	2.62 ± 0.62	
Preoperative CEA level			0.00^**^
< 5 ng/mL	40	1.86 ± 0.72	
≥ 5 ng/mL	15	3.13 ± 0.41	
Histologic grade			0.98
well differentiated	42	2.17 ± 0.89	
poorly differentiated	12	2.26 ± 0.80	
T classification			0.00^**^
T1 + T2	17	1.19 ± 0.54	
T3 + T4	37	2.65 ± 0.53	
TNM stage			0.38
I	4	1.57 ± 0.55	
II	14	2.28 ± 1.02	
III	28	2.18 ± 0.88	
IV	8	2.37 ± 0.51	
Lymphatic invasion			0.52
Negative	21	2.12 ± 0.91	
Positive	33	2.24 ± 0.84	
Distant metastasis			0.88
Negative	45	2.16 ± 0.92	
Positive	9	2.33 ± 0.50	

^a^54 fresh tissue samples from June 2018 to December 2018, ^b^protein level in Tumor tissues/protein level in Adjacent tissues (mean ± SD),**p ≤ 0.01

**Table 2:** Univariate and multivariate analysis of overall survival in CRC patients. (Cox proportional hazards regression model)

Parameters (130 CRC patients)^a^	Univariate analysis			Multivariate analysis		
	HR	95%CI	*p* value	HR	95%CI	*p* value
Age (≦60/> 60)	0.70	0.34–1.41	0.32			
Gender (Male/Female)	1.13	0.57–2.22	0.73			
Histologic grade (well/poor)	0.66	0.29–1.51	0.33			
Tumor size (cm) (< 5/≧5)	1.78	0.90–3.51	0.10			
CEA (ng/ml) (< 5/≧5)	1.09	0.75–1.60	0.64			
Lymphatic invasion (Positive/Negative)	1.95	1.04–3.67	0.04^*^	1.52	0.52–4.43	0.03^*^
Distant metastasis (Positive/Negative)	2.03	1.09–3.78	0.03^*^	1.65	0.62–4.38	0.01^**^
T classification (T3 + T4/T1 + T2)	2.65	1.20–5.85	0.02^*^	1.80	0.67–4.84	0.02^*^
TNM (III, IV/I, II)	2.49	1.24–5.00	0.01^**^	2.12	1.13–3.98	0.02^*^
JARID1B protein expression (High/Low)	2.29	1.41–3.73	0.00^**^	2.53	1.50–4.27	0.00^**^

^a^Immunohistochemical results of 130 paraffin-embedded tissues from December 2012 to December 2017, *p ≤ 0.05,**p ≤ 0.01

### JARID1B promoted the proliferation and tumorigenesis of CRC in vivo and in vitro

**Fig. 2:**
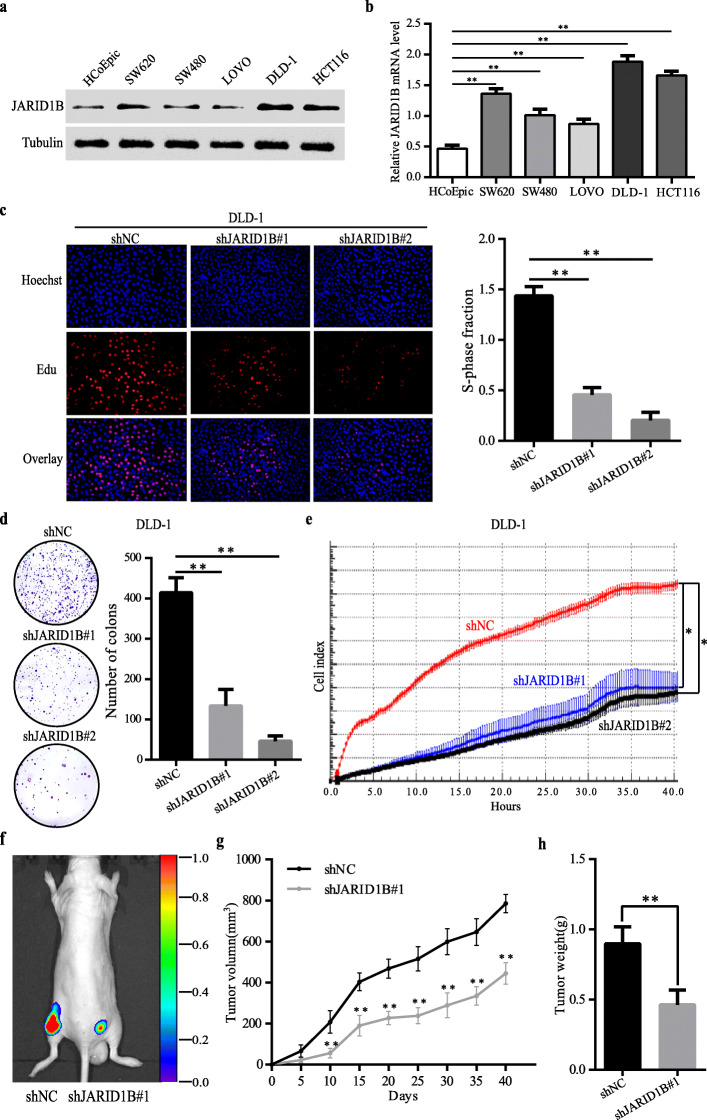
Downregulation of JARID1B expression led to decrease CRC proliferation in vitro and in vivo. **a**, **b** Western blot and qRT-PCR analysis of JARID1B protein expression in human nonmalignant cell line and CRC cell lines. **c**, **d**, **e** Cells proliferation capacities were detected by EdU, colony formation assay and RTCA assays in CRC DLD-1-cells transfected with the shJARID1B#1/#2 plasmid. **f** In-vivo tumor formation was examined by subcutaneously injecting CRC DLD-1-shNC (left) or CRC DLD-1-shJARID1B#1 (right) cells into the flanks of nude mice. Representative images obtained by an IVIS in-vivo imaging station after inoculation were shown. Corresponding tumor growth curves were obtained (**g**). **h** Tumor weight was counted at 40 days, JARID1B knockdown inhibited tumor proliferation.*p < 0.05, **p < 0.01

To investigate the potential biological function of JARID1B in CRC development, we initially examined JARID1B expression levels in CRC cells (SW620, HCT116, LOVO, SW480, DLD-1) and the non-malignant cell line (HCoEpic) by qRT-PCR and western blotting. The results showed that JARID1B expression was notably elevated in CRC cells (Fig. 2a, b). As shown in Table 1, the high JARID1B expression group demonstrated a larger tumour size, so we speculate that JARID1B may be functional in CRC proliferation. Next, we investigated the relationship between JARID1B expression and CRC cell proliferation. 5-ethynyl-20-deoxyuridine (EdU), colony formation and real-time cellular analysis (RTCA) assays revealed that the proliferation capacity of DLD-1 cells with short hairpin RNA (shRNA)-mediated JARID1B knockdown was markedly lower than that of the control group (Fig. 2c, d, e). In HCT116 where JARID1B was obviously increased as like DLD-1 cell lines, JARID1B knockdown also resulted in decreased cell proliferation (Figure S1). In contrast, JARID1B overexpression significantly enhanced the proliferation ability of LOVO cells (Figure S2). Furthermore, an in vivo experiment showed that the JARID1B knockdown group had smaller volumes and lower weights than the control group (Fig. 2f, g, h). That is, knockdown of JARID1B significantly inhibited tumour size and weight in vivo. These data demonstrated that JARID1B promotes CRC cell proliferation in vivo and in vitro.

### JARID1B influenced CRC proliferation by the Wnt/β-catenin signalling pathway

**Fig. 3:**
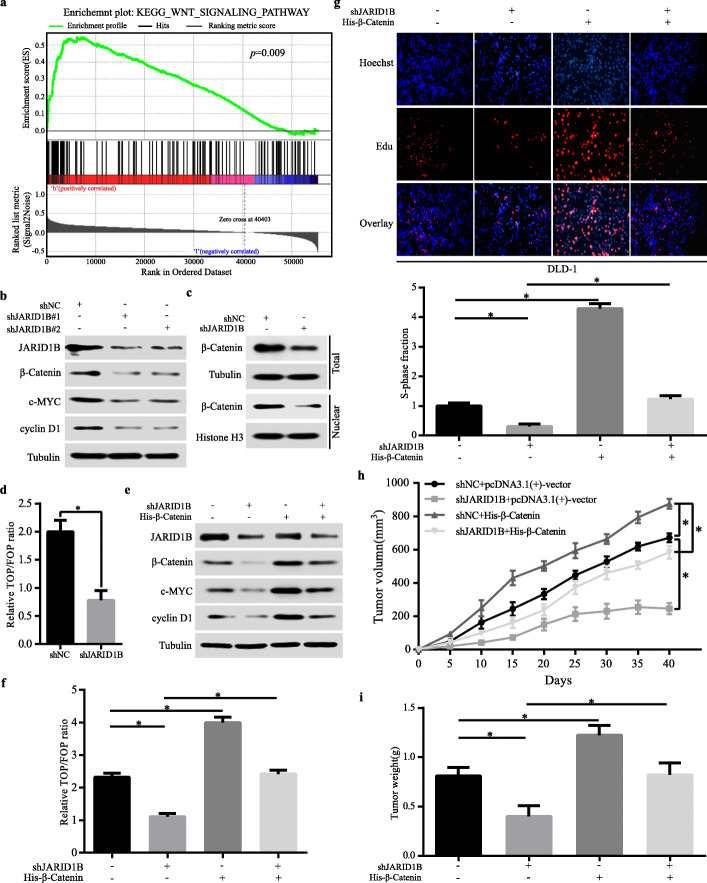
Stable knockdown of JARID1B repressed CRC proliferation via Wnt/β-catenin signaling pathway. **a** Gene set enrichment analysis of JARID1B based on TCGA COAD RNA Seq dataset showed that JARID1B expression was positively correlated with Wnt/β-catenin signaling pathway. **b** Western blot detected JARID1B, β-catenin c-MYC and cyclinD1 expression in DLD-1 cells transfected with shNC or shJARID1B. **c** The total and nuclear protein levels of β-catenin were assessed by western blotting in DLD-1 cells transfected with shJARID1B or shNC. **d** The inhibitive effect of JARID1B downregulation on Wnt/β-catenin pathway was detected by TOP-Flash luciferase reporter assay. **e** Western blot analysis showed the effects on c-MYC, cyclinD1 expression in DLD-1 cells when both JARID1B silencing and β-catenin restoration. **f** TOP-Flash luciferase reporter assay showing that β-catenin overexpression rescued the decreased Wnt/β-catenin pathway activity of DLD-1-shJARID1B cells. **g**, **h**, **i** EdU and in-vivo tumor formation assays showed that over-expression of ectopic β-catenin significantly rescued cells proliferation in DLD-1- shJARID1B cells. *p < 0.05

In subsequence, we explored the mechanism how JARID1B regulated CRC cell proliferation. Studies have indicated that the Wnt signalling pathway played an important role in CRC cells proliferation. Therefore, we speculated that JARID1B might regulate CRC cells proliferation by the Wnt/β-catenin signalling pathway. First, based on the TCGA COAD RNA expression dataset, Gene Set Enrichment Analysis (GSEA) revealed that JARID1B expression was positively correlated with Wnt signalling pathway (Fig. 3a). Second, we investigated the effect of JARID1B on the activation of the Wnt/β-catenin pathway in CRC cells. In DLD-1 cells with JARID1B knockdown, we observed that β-catenin, c-MYC and cyclin D1 were decreased (Fig. 3b). Knockdown of JARID1B significantly decreased total and nuclear β-catenin expression (Fig. 3c). Consistently, the TOP-Flash luciferase assay revealed that knockdown of JARID1B inhibited Wnt/β-catenin pathway activity, proving that JARID1B played a key role in activation of the Wnt/β-catenin signalling pathway (Fig. 3d). In contrast, JARID1B over-expression significantly increased c-MYC, cyclin D1 and total/nuclear β-catenin in LOVO cells (Figure S3a, b, c). These results indicated that the Wnt/β-catenin signalling pathway was regulated by JARID1B.

Next, we would like to validate that cells proliferation mediated by JARID1B depended on the Wnt/β-catenin signalling pathway. Thus, we increased β-catenin expression in JARID1B-knockdown DLD-1 cells, showing that β-catenin over-expression significantly increased β-catenin, c-MYC and cyclin D1 in JARID1B–knockdown DLD-1 cells, demonstrating β-catenin could rescue the decreased Wnt/β-catenin pathway activity induced by knockdown of JARID1B (Fig. 3e, f). Moreover, β-catenin over-expression could rescue the proliferation ability of DLD-1 cells with knockdown of JARID1B in vivo and in vitro (Fig. 3g, h, i). Conversely, we suppressed β-catenin expression in JARID1B-overexpressing LOVO cells, showing that β-catenin knockdown inhibited β-catenin, c-MYC and cyclin D1 expression and decreased Wnt/β-catenin pathway activated by JARID1B over-expression (Figure S3d, e). The enhanced proliferation ability caused by JARID1B over-expression in LOVO cells was markedly decreased by the knockdown of β-catenin (Figure S3f, g). However, JARID1B regulated CRC proliferation by the Wnt/β-catenin pathway.

### JARID1B significantly inhibited CDX2 expression in CRC

**Fig. 4:**
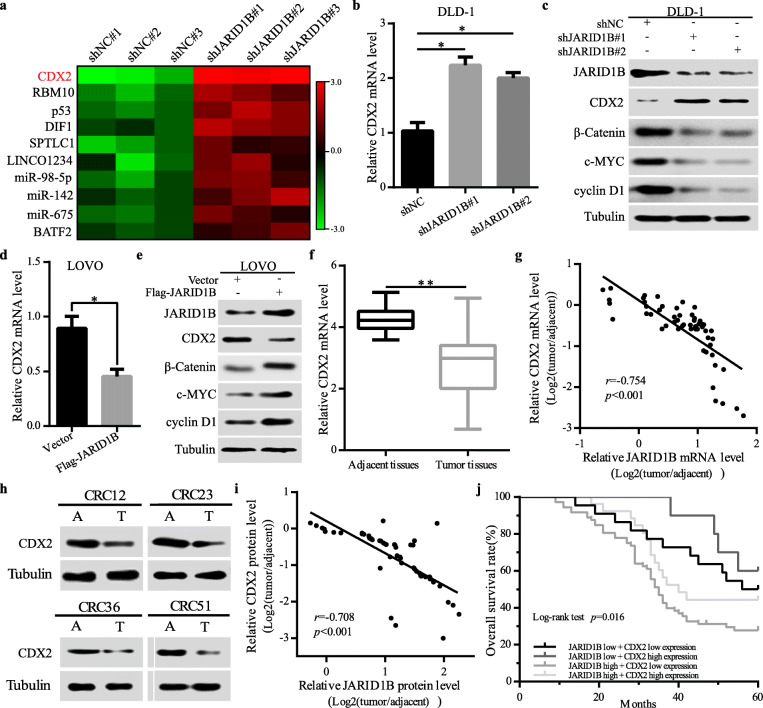
JARID1B inhibited CDX2 expression in CRC cells. **a** The microarray profiling of gene expression in DLD-1-shJARID1B cells. **b**, **c** qRT-PCR and western blot detected CDX2, β-catenin, c-MYC, cyclinD1 expression in DLD-1 cells transfected with shNC or shJARID1B. **d**, **e** qRT-PCR and western blot detected CDX2 expression in LOVO cells transfected with vector or Flag-JARID1B. **f**, **h** Western blot analysis and qRT-PCR of CDX2 protein levels in CRC tissues and in paired adjacent tissues. **g**, **i** Scatter plots showed a negative correlation between JARID1B and CDX2 at the mRNA and protein levels in fresh 54 CRC samples. **j** Kaplan-Meier analysis for 130 CRC patients follow-up data showed that the prognosis was the poorest for patients with both high JARID1B expression and low CDX2 expression. *p < 0.05, **p < 0.01

We further explored how JARID1B regulated Wnt/β-catenin signalling in CRC cells. It has been reported that CDX2 knockdown promotes the proliferation of colorectal cancer cells via Wnt/β-catenin signalling. Thus, we speculated that JARID1B might regulate the expression of CDX2 to enhance the activity of Wnt/β-catenin signalling in CRC cells. Given the role of JARID1B in the epigenetic regulation of transcription, we first performed RNA-seq to identify potential JARID1B target genes involved in cell proliferation. The results showed that CDX2 was one of the most notably upregulated transcripts when JARID1B was knocked down (Fig. 4a). Next, qRT-PCR revealed that downregulation of JARID1B significantly increased CDX2 mRNA (Fig. 4b). Western blotting results showed that downregulation of JARID1B expression significantly increased the expression levels of CDX2, while the expression levels of β-catenin, c-MYC and cyclin D1 were decreased (Fig. 4c). Conversely, JARID1B overexpression decreased the levels of CDX2 mRNA expression (Fig. 4d). Western blotting results showed that upregulation of JARID1B expression significantly decreased the expression levels of CDX2, while the expression levels of β-catenin, c-MYC and cyclin D1 were increased (Fig. 4e). Furthermore, we examined CDX2 in 54 CRC tissue samples by western blotting and qRT-PCR. The results showed that the expression level of CDX2 was significantly higher in adjacent tissues than in tumour tissues (Fig. 4f, h). Finally, the statistical analysis results revealed that CDX2 expression was negatively correlated with JARID1B expression in CRC tissues (Fig. 4g, i). Consistently, Kaplan-Meier analysis indicated that patients with both high JARID1B expression and low CDX2 expression in 130 immunohistochemical results predicted the worst prognosis (Fig. 4i). Collectively, these data suggest that JARID1B negatively regulates CDX2 expression to increase Wnt/β-catenin signalling activity in CRC.

### CDX2 was the key protein for JARID1B-mediated Wnt/β-catenin signalling in CRC cells

**Fig. 5:**
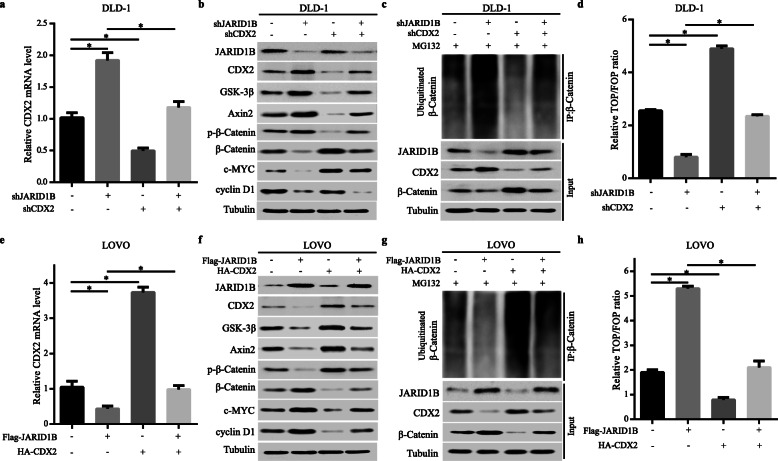
CDX2 was required for JARID1B-mediated Wnt/β-catenin signaling in CRC cells. **a** mRNA levels of CDX2 were detected by qRT-PCR. The knockdown of CDX2 expression dramatically inhibits the increase of CDX2 expression in DLD-1-shJARID1B cells. **b** Western blot analysis showed that the knockdown of CDX2 expression markedly rescused the increase of GSK-3β, Axin2 and p-β-catenin expression and the decrease of β-catenin, c-MYC and cyclinD1 expression induced by JARID1B knockdown. **c** Ubiquitinated β-catenin levels when JARID1B and CDX2 knockdown in DLD-1 cells. **d** Silencing CDX2 attenuated the loss of downregulating JARID1B on Wnt/β-catenin as observed by TOP-Flash luciferase assay. **e** The upregulation of CDX2 mRNA expression markedly inhibited the decrease in CDX2 expression observed in JARID1B-overexpression LOVO cells. **f** Western blot analysis showed that the levels of JARID1B overexpression and CDX2 upregulation and their effects on GSK-3β, Axin2, β-catenin, c-MYC and cyclinD1 expression in LOVO cells. **g** Ubiquitinated β-catenin levels when JARID1B and CDX2 over-expressed in LOVO cells. **h** TOP-Flash luciferase reporter assay showed the overexpression of ectopic CDX2 attenuated the increase in Wnt/β-catenin activitiy in JARID1B-overexpression LOVO cells.*p < 0.05

To further validate whether JARID1B activated the Wnt/β-catenin signalling pathway by regulating CDX2 expression. After we first decreased the expression of CDX2 in JARID1B–knockdown DLD-1 cells, CDX2, GSK-3β, Axin2 and p-β-catenin, β-catenin, c-MYC and cyclin D1 expression levels were observed. The qRT-PCR results showed that knockdown of CDX2 inhibited the increase in CDX2 mRNA expression induced by JARID1B knockdown (Fig. 5a). Western blotting data showed that CDX2 knockdown decreased GSK-3β, Axin2 and p-β-catenin expression and markedly rescued the changes in β-catenin, c-MYC and cyclin D1 expression levels caused by JARID1B (Fig. 5b). After β-catenin IP among different groups ensured that IP worked and the differences between samples were real (Figure S4), Co-IP showed that JARID1B knockdown increased the ubiquitinated β-catenin level, which was rescued when CDX2 expression was knocked down (Fig. 5c). Simultaneously, the TOP-Flash luciferase assay revealed that the reduced Wnt/β-catenin signalling activity induced by JARID1B knockdown was partly abolished by the knockdown of CDX2 (Fig. 5d). In contrast, upregulation of CDX2 inhibited the JARID1B overexpression-induced increase in LOVO cells (Fig. 5e, f, g, h). These results demonstrated that CDX2 was required for the JARID1B-mediated Wnt/β-catenin signalling pathway in CRC cells.

### JARID1B regulated CDX2 expression through demethylation of H3K4me3

**Fig. 6:**
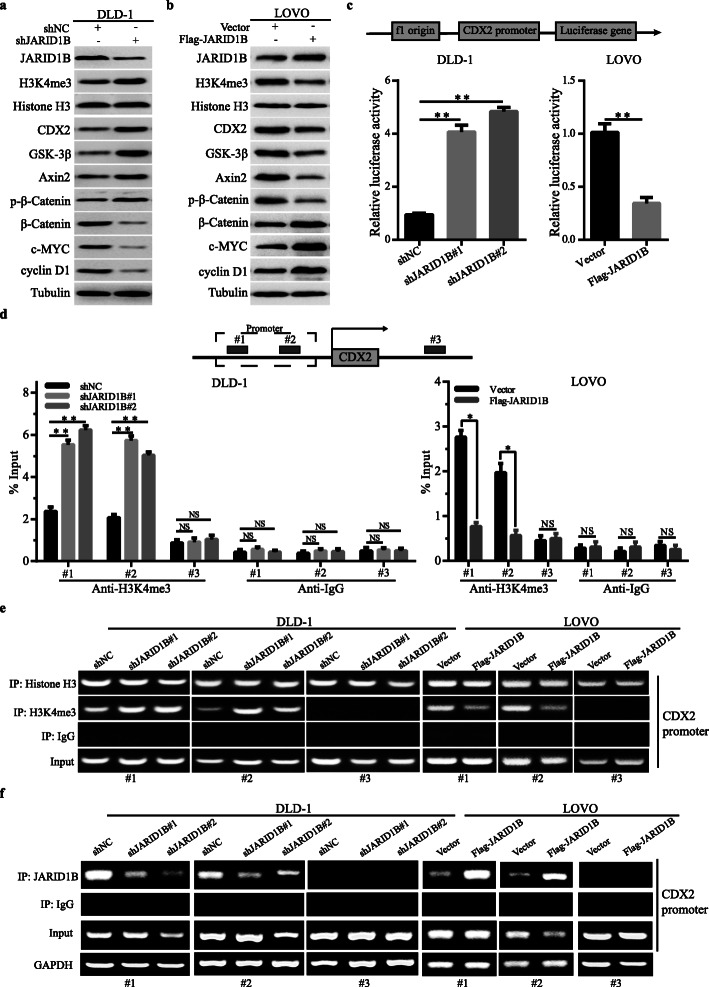
JARID1B demethylated H3K4me3 at the CDX2 promoter. **a**, **b** Western blot analysis was performed to detect the expression of CDX2, H3K4me3, Histone H3, GSK-3β, Axin2, p-β-catenin, β-catenin, c-MYC and cyclinD1 in DLD-1-shJARID1B and control DLD-1 cells, LOVO-JARID1B and control LOVO cells. **c** CDX2 promoter reporter luciferase assay using CRC cells transfected with the shJARID1B and shNC plasmid. Western blot was used to detect JARID1B, H3K4me3, CDX2, β-catenin and c-MYC expression in DLD-1 cells transfected with shNC or shJARID1B and in LOVO cells transfected with Vector or Flag-JARID1B. **d** Schematic representation of the CDX2 promoter and the two predicted H3K4me3 binding elements in the promoter region of the CDX2 gene. Quantitative chromatin immunoprecipitation (qChIP) assays were performed in shJARID1B, control DLD-1 cells, JARID1B-overexpression and control LOVO cells. **e** When JARID1B knockdown, ChIP qPCR of H3K4me3 to the CDX2 promoter region was up-regulated. In contrast, ChIP qPCR of H3K4me3 to the CDX2 promoter region was down-regulated when JARID1B over-expression, normalized by input. **f** ChIP qPCR of JARID1B to the CDX2 promoter region was down-regulated when JARID1B knockdown. In contrast, ChIP qPCR of JARID1B to the CDX2 promoter region was up-regulated when JARID1B over-expression, normalized by input. GAPDH as an internal control. *p < 0.05, **p < 0.01, NS: no significant

We then explored how JARID1B regulated CDX2 expression at the transcriptional level. Studies have shown that JARID1B affects histone H3 lysine 4 (H3K4) demethylase and exhibits a strong transcriptional repression function. We explored whether JARID1B demethylates H3K4me3 at the promoter of CDX2 in CRC cells. We first decreased the expression of JARID1B and then observed the H3K4me3 protein expression levels and CDX2 promoter activity. Western blotting data showed that JARID1B knockdown markedly increased H3K4me3, CDX2, GSK-3β, Axin2 and p-β-catenin expression and decreased β-catenin, c-MYC and cyclin D1 expression (Fig. 6a). Simultaneously, a luciferase reporter gene assay showed that JARID1B downregulation increased CDX2 promoter activity (Fig. 6c). In contrast, overexpression of JARID1B decreased H3K4me3 protein expression and CDX2 promoter activity (Fig. 6b, c). In addition, we tested whether JARID1B expression was correlated with H3K4me3 modification at the CDX2 gene promoter in CRC cells. Moreover, the ChIP assay revealed that JARID1B knockdown increased H3K4me3 levels at CDX2 in DLD-1 cells (Fig. 6d), while overexpression of JARID1B decreased H3K4me3 levels at CDX2 in LOVO cells (Fig. 6d). Consistently, the ChIP qPCR assay revealed that JARID1B was recruited to CDX2 promoter, resulting in trimethylation of H3K4 in this region (Fig. 6e and f). After combining all the experimental results, we revealed a new important mechanism by which JARID1B regulated CDX2 expression through H3K4me3 to indirectly activate the Wnt/β-catenin pathway, leading to increased CRC proliferation.

## Discussion

Tumour proliferation plays a crucial role in the development of CRC. Studies have confirmed that the proliferation of CRC cells is significantly correlated with the abnormal expression of histone demethylase. JARID1B plays an important role in cell fate decisions, cancer progression, and stem cell self-renewal [25, 26]. Evidence is emerging that JARID1B contributes to the epigenetic plasticity that underlies malignant transformation [8]. Studies have also shown that JARID1B is overexpressed in numerous cancers [27], and JARID1B overexpression is associated with a poor prognosis in breast and prostate cancers [28, 29]. Studies have shown that the histone demethylase JARID1B is associated with CRC cell growth [14]. In this study, we explored the role of JARID1B in CRC proliferation. We found that JARID1B is significantly elevated in CRC, and overexpression of JARID1B protein expression resulted in significantly shorter overall survival. Furthermore, high JARID1B expression was closely associated with shorter overall survival in CRC patients. In addition, we provided evidence demonstrating that JARID1B promotes CRC cell proliferation in vivo and in vitro.

Next, we further explored the underlying mechanism by which JARID1B regulates CRC proliferation. The present studies have demonstrated that abnormal activation of the Wnt/β-catenin signalling pathway is an important cause of CRC [30]. Studies on the molecular mechanism of tumorigenesis and development related to the Wnt/β-catenin signalling pathway have always focused on four parts: receptors on the cell membrane, inhibitors or activators in the cytoplasm, transcription factors in the nucleus and downstream target genes of β-catenin [31]. In hypopharyngeal squamous cell carcinoma, JARID1B inhibits cell proliferation by activating β-catenin signalling [32]. Moreover, in hepatocellular carcinoma, basil polysaccharide was found to attenuate metastasis of rat hepatocellular carcinoma, simultaneously resulting in the downregulation of both JARID1B and β-catenin [33]. In line with these findings, we found a novel mechanism by which JARID1B regulates CRC proliferation via Wnt/β-catenin signalling. First, we found that the Wnt/β-catenin signalling pathway is the downstream pathway of JARID1B, and JARID1B levels were positively correlated with Wnt/β-catenin activity in CRC cells. Second, as JARID1B expression increased, the expression of β-catenin, c-MYC and cyclin D1 decreased, whereas JARID1B upregulation again had the opposite effect in CRC cells. Finally, β-catenin overexpression significantly inhibited the decrease in c-MYC and cyclin D1 expression in JARID1B–knockdown CRC cells and rescued the decreased Wnt/β-catenin pathway activity induced by downregulating JARID1B, rescued the proliferation ability in CRC cells with JARID1B downregulation in vivo and in vitro. In contrast, downregulation of β-catenin had the opposite results in JARID1B-overexpressing CRC cells. These results revealed a mechanism by which JARID1B contributes to CRC proliferation by activating the Wnt/β-catenin pathway.

Our current study also highlights the detailed mechanism by which the Wnt/β-catenin pathway is regulated by JARID1B in CRC cells. Caudal-type homeobox transcription factor 2 (CDX2), an essential intestine-specific regulator, is involved in the development and differentiation of intestinal epithelial cells and regulates the balance between cell proliferation and differentiation [34]. In the present study, many studies revealed that the CDX2 expression level was associated with CRC cell proliferation and poor prognosis for patients with CRC [35]. Recent evidence indicated that CDX2 inhibited the progression of CRC by suppressing the Wnt/β-catenin signalling pathway and transactivating GSK-3β and Axin2 expression [36]. Furthermore, CDX is a crucial member of the Wnt/β-catenin signalling pathway, and its expression is decreased in the liver metastasis of CRC [37]. Here, we revealed a novel mechanism by which JARID1B regulates the Wnt/β-catenin signalling pathway by inhibiting CDX2 expression. First, we found that CDX2 might be the most relevant gene in transcriptome sequencing technology of JARID1B-downregulated CRC cells. We confirmed the negative correlation between JARID1B and CDX2 expression levels by cell experiments and detection of clinical tissues. Finally, we explored the mechanism by which JARID1B regulates CDX2 expression in CRC cells. A previous study showed that JARID1B enables H3K4me3 demethylation, and the depletion of JARID1B has been shown to specifically inhibit H3K4 demethylation and suppress CRC cell growth [11, 14].

Therefore, we proposed the underlying mechanism: JARID1B regulates CDX2 expression through H3K4me3. This conclusion was based on the following observations: on the one hand, JARID1B knockdown markedly increased H3K4me3 protein expression and CDX2 promoter activity; on the other hand, JARID1B overexpression decreased H3K4me3 levels and CDX2 promoter activity. However, further studies are needed to clarify whether there were some proteins as a complex participating in the mechanism between JARID1B and H3K4me3, and further study on the mechanism of how JARID1B affects the activation of CDX2 promoter, providing new ideas for further understanding the pathogenesis and development of CRC.

## Conclusions

In summary, our study demonstrated that JARID1B acted as an oncogene to promote the CRC progression. JARID1B inhibited CDX2 transcription level by demethylation of H3K4me3, which was effected at the CDX2 promoter region. Following CDX2 low expression, GSK-3β and Axin2 expression levels were decreased, and further phosphorylated β-catenin level was inhibited. So that less β-catenin was bound by ubiquitination to degrade, and more β-catenin was transported into the nucleus, which caused downstream target genes (c-MYC and cyclinD1) to be activated (Fig. 6e). Finally, CRC cells proliferation was accelerated. Our results uncovers the diverse role of JARID1B in cell biology and function of JARID1B in cancer development, which extends foundation for the development of new anti-cancer therapeutic strategies.

## Supplementary information

Additional file 1: **Table S1.** Primers and shRNA target sequences. (DOC 33 kb)

Additional file 2: **Figure S1.** JARID1B knockdown in HCT116 resulted in decreased CRC proliferation. a, b Western blot showed the knockdown efficiency of JARID1B in DLD-1 and HCT116. c, d, e Cells proliferation capacities were detected by EdU, colony formation assay and RTCA assays in CRC HCT116 cells transfected with the shJARID1B#1/#2 plasmid. *p < 0.05, **p < 0.01. (TIF 15203 kb)

Additional file 3: **Figure S2.** JARID1B overexpression promoted CRC cells proliferation. a, b Cells proliferation capacities as detected by Colony formation and EdU in LOVO cells transfected with Vector or Fla-JARID1B.**p < 0.01. (TIF 4842 kb)

Additional file 4: **Figure S3.** JARID1B overexpression promoted CRC cells proliferation by inhibiting CDX2 expression. a Western blot detected JARID1B, β-catenin, c-MYC and cyclinD1expression in LOVO cells transfected with Vector or Flag-JARID1B. b The total and nuclear protein levels of β-catenin were assessed by western blotting in LOVO cells transfected with Vector or Flag-JARID1B. c The effect of JARID1B overexpression on Wnt/β-catenin pathway was detected by TOP-Flash luciferase reporter assay. d Western blotting analysis showed downregulation of β-catenin attenuated the increased expression of β-catenin, c-MYC and cyclinD1 in LOVO-JARID1B cells. e TOP-Flash luciferase reporter assay showing that β-catenin knockdown rescued the increased Wnt/β-catenin pathway activity of LOVO-JARID1B cells. g, h Edu assay results showed that β-catenin knockdown significantly inhibited the increase of cells proliferation in LOVO-JARID1B cells. *p < 0.05. (TIF 12820 kb)

Additional file 5: **Figure S4.** β-catenin IB in β-catenin IP ensured that IP worked and the differences between samples were real. a In DLD-1, β-catenin IP while there were different groups including shJARID1B, shCDX2 and shJARID1B/shCDX2. b In LOVO, β-catenin IP while there were different groups including Flag-JARID1B, HA-CDX2 and Flag-JARID1B/HA-CDX2. (TIF 5295 kb)

## Data Availability

The datasets supporting the conclusions of this article are included within the article and its additional files.

## Abbreviations

JARID1B: Jumonji AT-rich interactive domain 1B
CRC: Colorectal cancer
EdU: 5-ethynyl-20-deoxyuridine
RTCA: Real Time Cellular Analysis
ChIP: Chromatin immunoprecipitation Assay
GSEA: Gene Set Enrichment Analysis
H3K4: Histone H3 lysine4
CDX2: Caudal-type homeobox transcription factor 2
qRT-PCR: quantitative reverse transcriptase PCR
Ub: Ubiquitin
Co-IP: Co-immunoprecipitation
IHC: Immunohistochemistry
