# Supplementary material for: JARID1B promotes colorectal cancer proliferation and Wnt/β-catenin signaling via decreasing CDX2 level
Source: Cell Commun Signal. 2020 Oct 27;18:169. doi: 10.1186/s12964-020-00660-4 (PMC7590656; doi:10.1186/s12964-020-00660-4)
Supplement: Supplementary file 2 — Table S1. Primers and shRNA target sequences. (DOC 33 kb) [file 12964_2020_660_MOESM2_ESM.doc]

**Supplementary Table.1** Primers and shRNA target sequences

| name | sequence | accession number | Enzyme |
| --- | --- | --- | --- |
| Primers for real-time PCR: |  |  |  |
| JARID1B sense | 5'-AGCCAAACCTGACCACAGAC-3′ | NM_001314042.1 |  |
| JARID1B antisense | 5'- TTCTGGCTTCCGTTGTCTCC-3′ | NM_001314042.1 |  |
| CDX2 sence | 5'- GAGTGGTGTACACGGACCAC-3′ | NM_001265.6 |  |
| CDX2 antisence | 5'- CCAGATTTTAACCTGCCTCTCAG -3′ | NM_001265.6 |  |
| GAPDH sense | 5’-CTTCATTGACCTCAACTACA-3’ | NM_001256799.2 |  |
| GAPDH antisense | 5’-ACTCCACGACATACTCAGC-3’ | NM_001256799.2 |  |
| Primers for plasmids construct: |  |  |  |
| pcDNA3.1(+)-HA-CDX2  sense | 5'-CGGGATCCGCCACCATGTCCATTTCTGGGACGCTT-3' |  | BamHI |
| pcDNA3.1(+)-HA-CDX2 antisense | 5'-GGAATTCTTACTCTTTGCCCTGCTCCTTAT-3' |  | EcoRI |
| pcDNA3.1(+)-Flag-JARID1B sense | 5’-ACGGGCCCTCTAGACTCGAGCGCCACCATGAA  GCTCTCCCTGGTGGC-3’ |  | BamHI |
| pcDNA3.1(+)-Flag-JARID1B antisense | 5’-AGTCACTTAAGCTTGGTACCGACAACTCATCTT  TTTCTGCTGTATC-3’ |  | EcoRI |
| pcDNA3.1(-)-His-β-catenin  sense | 5'-GGAATTCGCCACCATGCAGATCTTCGTGAAAACC-3' |  | BamHI |
| pcDNA3.1(-)-His-β-catenin antisense | 5'-CCCAAGCTTACAGCCACCCCTCAGGCGCAG-3' |  | HindII |
| The target sites of shRNA: |  |  |  |
| shJARID1B#1 | GACCCCTTCGCTTTCATCCA |  |  |
| shJARID1B#2 | GGAGGCCCAAACTCGTGTAA |  |  |
| shJARID1B#3 | cacatgtggagaggaagatc |  |  |
| shCDX2 | GGGAGGACTGGAATGGCTAC |  |  |
| shNC | TTCTCCGAACGTGTCACGT |  |  |
