# Supplementary figures and images for: JARID1B promotes colorectal cancer proliferation and Wnt/β-catenin signaling via decreasing CDX2 level
Source: Cell Commun Signal. 2020 Oct 27;18:169. doi: 10.1186/s12964-020-00660-4 (PMC7590656; doi:10.1186/s12964-020-00660-4)

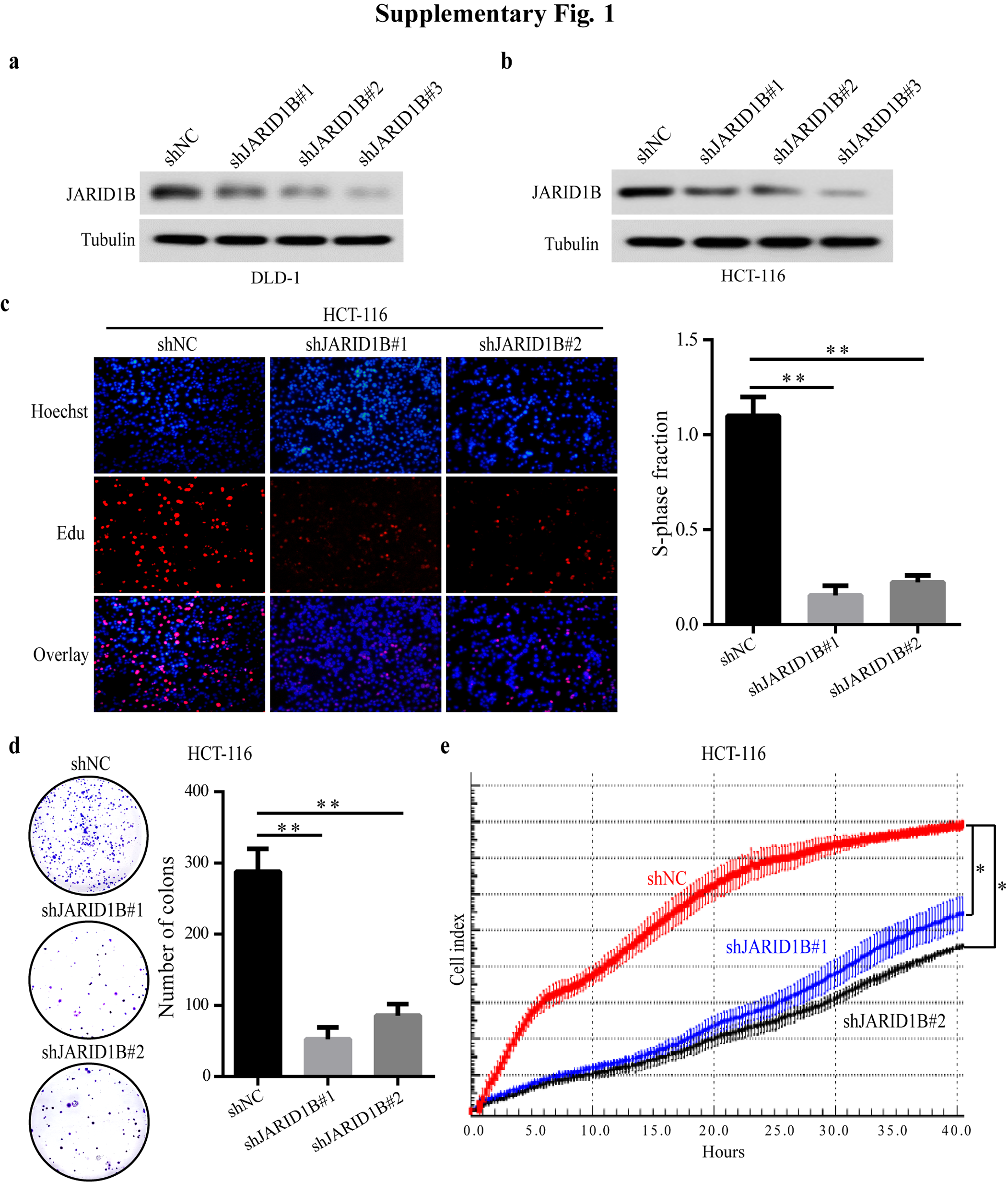

Supplement: Supplementary file 3 — Figure S1. JARID1B knockdown in HCT116 resulted in decreased CRC proliferation. a, b Western blot showed the knockdown efficiency of JARID1B in DLD-1 and HCT116. c, d, e Cells proliferation capacities were detected by EdU, colony formation assay and RTCA assays in CRC HCT116 cells transfected with the shJARID1B#1/#2 plasmid. *p < 0.05, **p < 0.01. (TIF 15203 kb) [file 12964_2020_660_MOESM3_ESM.tif]

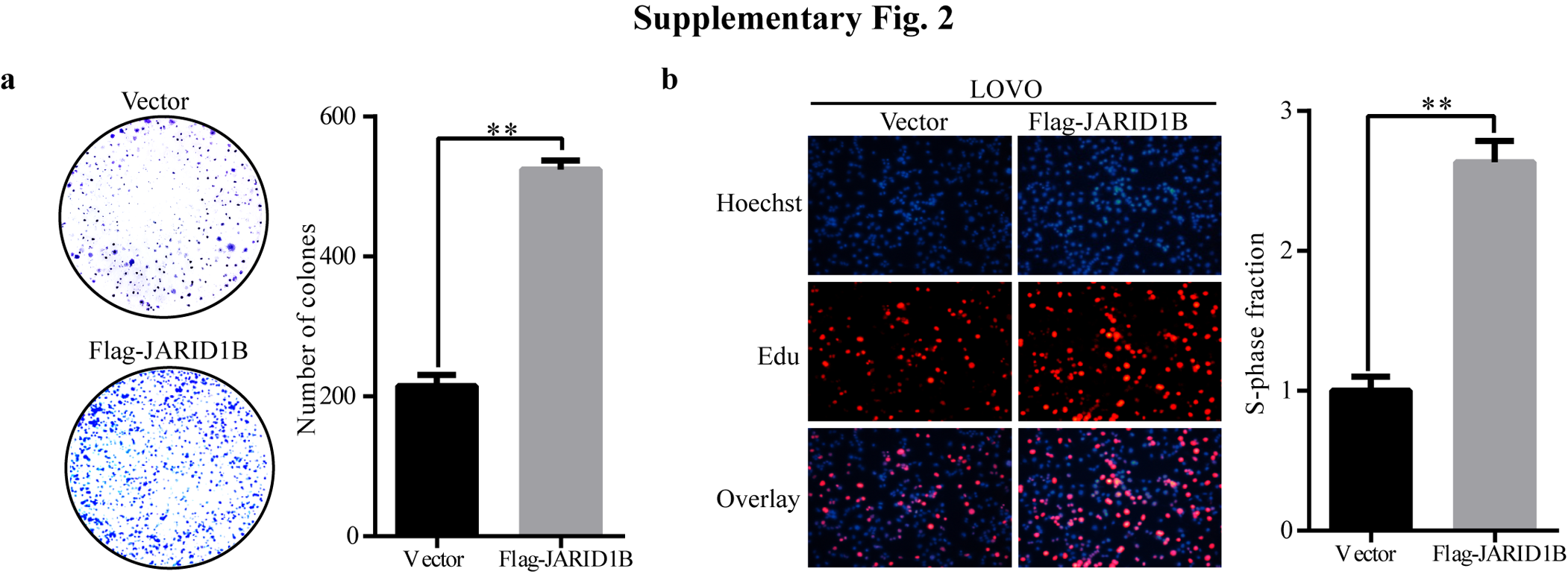

Supplement: Supplementary file 4 — Figure S2. JARID1B overexpression promoted CRC cells proliferation. a, b Cells proliferation capacities as detected by Colony formation and EdU in LOVO cells transfected with Vector or Fla-JARID1B.**p < 0.01. (TIF 4842 kb) [file 12964_2020_660_MOESM4_ESM.tif]

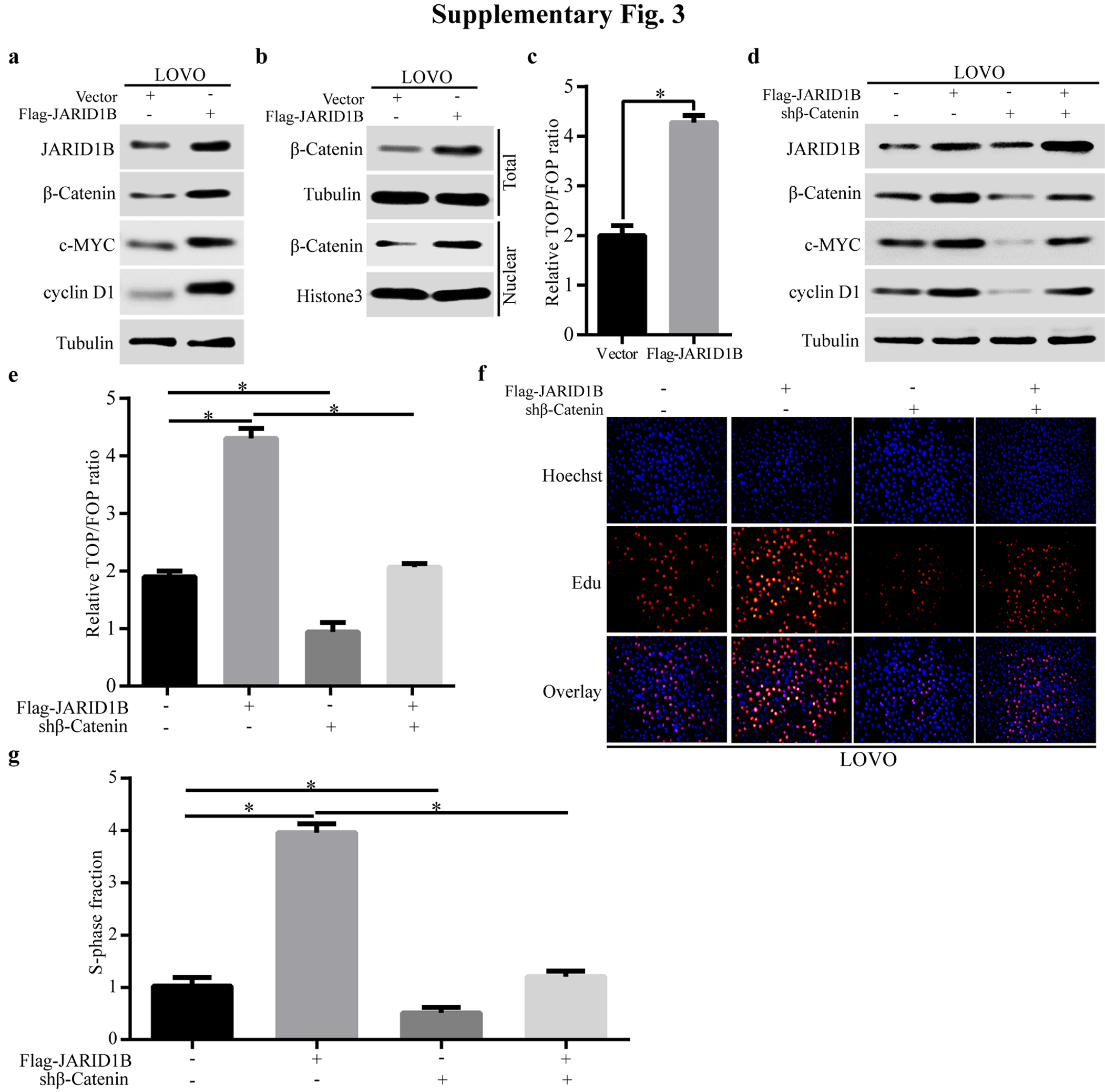

Supplement: Supplementary file 5 — Figure S3. JARID1B overexpression promoted CRC cells proliferation by inhibiting CDX2 expression. a Western blot detected JARID1B, β-catenin, c-MYC and cyclinD1expression in LOVO cells transfected with Vector or Flag-JARID1B. b The total and nuclear protein levels of β-catenin were assessed by western blotting in LOVO cells transfected with Vector or Flag-JARID1B. c The effect of JARID1B overexpression on Wnt/β-catenin pathway was detected by TOP-Flash luciferase reporter assay. d Western blotting analysis showed downregulation of β-catenin attenuated the increased expression of β-catenin, c-MYC and cyclinD1 in LOVO-JARID1B cells. e TOP-Flash luciferase reporter assay showing that β-catenin knockdown rescued the increased Wnt/β-catenin pathway activity of LOVO-JARID1B cells. g, h Edu assay results showed that β-catenin knockdown significantly inhibited the increase of cells proliferation in LOVO-JARID1B cells. *p < 0.05. (TIF 12820 kb) [file 12964_2020_660_MOESM5_ESM.tif]

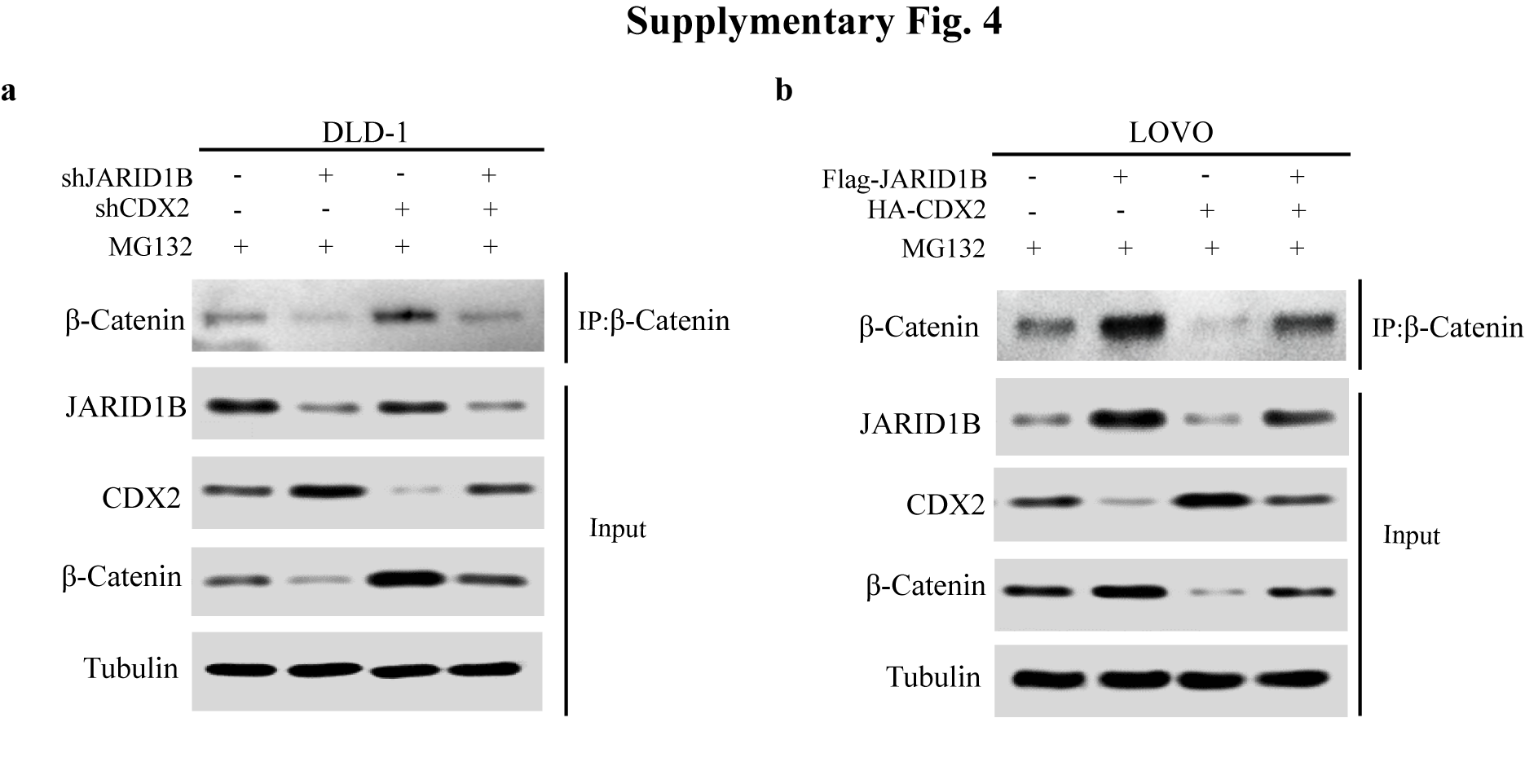

Supplement: Supplementary file 6 — Figure S4. β-catenin IB in β-catenin IP ensured that IP worked and the differences between samples were real. a In DLD-1, β-catenin IP while there were different groups including shJARID1B, shCDX2 and shJARID1B/shCDX2. b In LOVO, β-catenin IP while there were different groups including Flag-JARID1B, HA-CDX2 and Flag-JARID1B/HA-CDX2. (TIF 5295 kb) [file 12964_2020_660_MOESM6_ESM.tif]
